# The effect of facility characteristics on patient safety, patient experience, and service availability for procedures in non-hospital-affiliated outpatient settings: A systematic review

**DOI:** 10.1371/journal.pone.0190975

**Published:** 2018-01-05

**Authors:** Nancy F. Berglas, Molly F. Battistelli, Wanda K. Nicholson, Mindy Sobota, Richard D. Urman, Sarah C. M. Roberts

**Affiliations:** 1 Advancing New Standards in Reproductive Health, University of California, San Francisco (UCSF), Oakland, California, United States of America; 2 University of North Carolina, Chapel Hill, North Carolina, United States of America; 3 Rhode Island Hospital, Alpert Medical School at Brown University, Providence, Rhode Island, United States of America; 4 Brigham and Women’s Hospital, Harvard Medical School, Boston, Massachusetts, United States of America; Azienda Ospedaliero Universitaria Careggi, ITALY

## Abstract

**Background:**

Over recent decades, numerous medical procedures have migrated out of hospitals and into freestanding ambulatory surgery centers (ASCs) and physician offices, with possible implications for patient outcomes. In response, states have passed regulations for office-based surgeries, private organizations have established standards for facility accreditation, and professional associations have developed clinical guidelines. While abortions have been performed in office setting for decades, states have also enacted laws requiring that facilities that perform abortions meet specific requirements. The extent to which facility requirements have an impact on patient outcomes—for any procedure—is unclear.

**Methods and findings:**

We conducted a systematic review to examine the effect of outpatient facility type (ASC vs. office) and specific facility characteristics (e.g., facility accreditation, emergency response protocols, clinician qualifications, physical plant characteristics, other policies) on patient safety, patient experience and service availability in non-hospital-affiliated outpatient settings. To identify relevant research, we searched databases of the published academic literature (PubMed, EMBASE, Web of Science) and websites of governmental and non-governmental organizations. Two investigators reviewed 3049 abstracts and full-text articles against inclusion/exclusion criteria and assessed the quality of 22 identified articles. Most studies were hampered by methodological challenges, with 12 of 22 not meeting minimum quality criteria. Of 10 studies included in the review, most (6) examined the effect of facility type on patient safety. Existing research appears to indicate no difference in patient safety for outpatient procedures performed in ASCs vs. physician offices. Research about specific facility characteristics is insufficient to draw conclusions.

**Conclusions:**

More and higher quality research is needed to determine if there is a public health problem to be addressed through facility regulation and, if so, which facility characteristics may result in consistent improvements to patient safety while not adversely affecting patient experience or service availability.

## Introduction

The Institute of Medicine’s seminal reports, *To Err is Human* (1999) and *Crossing the Quality Chasm* (2002) brought national attention to concerns about patient safety in the health care system and led to efforts to study and improve safety across health care facility settings, primarily in hospitals [1, 2]. Around the same time, surgeries and procedures that had historically been performed solely in licensed hospitals transitioned to less resource intensive settings, including freestanding ambulatory surgery centers (ASCs), physician offices and clinics [3]. As of 2006, an estimated 53 million surgical and nonsurgical procedures were performed annually on an outpatient basis [3]. This migration of care raised important questions about patient safety and has led to efforts to study and improve patient experience in non-hospital health care settings as well. There has been increased attention to patient experience and outcomes in outpatient settings by academic researchers, professional associations, state legislatures, payors and private accrediting organizations.

Nonetheless, research on the effect of undergoing a procedure in a particular type of outpatient facility—ASC or physician office—has been limited. The question of differential risk by outpatient setting has primarily been raised within the field of cosmetic/plastic surgery, following public concerns about patient safety in offices in the 1990s and subsequent efforts to address concerns through state office-based surgery laws, facility accreditation, mandated reporting of adverse events, and quality improvement activities. The State of Florida’s adverse event registry, in particular, has been used by researchers to understand risk in physician offices [4–12]. Other researchers have used claims data to study differences in offices and ASCs, with particular attention to patient risk factors in each setting [13–15].

Since 2011, states have enacted an increasing number of laws that mandate specific requirements for the facilities in which abortions are performed [16]. Supporters of these laws maintain that facility regulations make abortion safer, despite the fact that abortion has a well-documented patient safety record over 40 years that meets or exceeds those of other outpatient procedures [17–19]. Research indicates that the challenges of complying with these laws have resulted in facility closures, dramatically reducing the availability of safe abortion services [20].

In 2016, the U.S. Supreme Court ruled against a Texas law mandating that abortion be performed in facilities licensed as ASCs and by physicians with local hospital admitting privileges. In its decision, the Court held that laws regulating the provision of abortion are unconstitutional if the burdens they impose are not balanced by proportional benefits. It also instructed future courts considering challenges to such laws to carefully assess whether the law is based on credible evidence, rather than relying on speculation or the judgement of a state agency or legislature [21]. This raises the critical question of what quality scientific evidence exists regarding the impact of facility requirements, both for abortion and other common outpatient procedures. To date, the methodological quality of the literature and the consistency of results across these studies have not been systematically assessed.

### Purpose of the study

In this study, we conduct a systematic review to examine the effect of facility type (ASC vs. office/clinic) and specific facility characteristics (e.g., facility accreditation, emergency response protocols, clinician qualifications, physical plant characteristics, other facility policies) on patient outcomes for procedures commonly performed in non-hospital-affiliated outpatient settings. We examine patient safety outcomes, as well as those related to patient experience and availability of services. We aim to identify and consolidate the existing body of research across medical procedures, and then assess the quality of the research and the consistency of findings across studies.

## Materials and methods

### Scope of review

The aim of the systematic review is to examine the impact of facility type and specific facility characteristics on patient safety, patient experience and service availability. We sought to answer the following two research questions:

Q1. What is the effect of facility type (ASC vs. office/clinic) on patient safety, patient experience and service availability for procedures in non-hospital-affiliated outpatient settings?
Q2. What is the effect of specific facility characteristics on patient safety, patient experience and service availability for procedures in non-hospital-affiliated outpatient settings?

For the second research question, we identified various types of requirements governing facility operations that appear in many accreditation standards and state laws, including those generally applicable to office-based surgeries and those specifically intended to regulate abortion providers [22]. We categorized these requirements according to their focus on facility accreditation, emergency response protocols, clinician qualifications, physical plant characteristics, and other facility policies and procedures (Table 1).

**Table 1:** Common facility requirements in non-hospital-affiliated outpatient settings, used to guide Q2 review.

Domain	Facility Requirements
Facility Accreditation	Facility accreditation by independent entity
Emergency Response Protocols	Hospital admitting privileges
	Transfer agreements with hospital and/or back-up physician
	Plan or protocol to facilitate patient transfers
Clinician Qualifications	Provider qualification beyond state licensing (e.g., specific board certification, specific residency training)
	Specific levels of nursing staff
Physical Plant Characteristics	Rooms in which procedures are performed
	Separate soiled & clean instrument sterilization rooms
	Separate recovery room
	Hall and/or door widths
	Emergency power
	Temperature and ventilation
	National Fire Protection Association (NFPA) compliance
Other Facility Policies & Procedures	Risk management (e.g., maintenance, infection control, disaster preparation)
	Quality assurance program
	Assessment of patient experience
	Peer review process

We conducted the review according to the Preferred Reporting Items for Systematic Reviews and Meta-Analyses (PRISMA) guidelines (S1 Table). We registered the study prospectively with the international registry for systematic reviews, PROSPERO (#CRD42016046872).

### Data sources and search strategy

We developed the search strategy in collaboration with a university reference librarian, who assisted with the selection of databases, development of search terms, and reference management. We searched the electronic databases EMBASE, PubMed (including MEDLINE) and Web of Science for relevant publications. The search strategy involved using each database’s controlled vocabulary (e.g., Medical Subject Heading (MeSH) terms for PubMed, Emtree for EMBASE) as well as a range of relevant keywords identified through the literature. We conducted separate searches for each of the research questions. We limited all searches to articles published in the English language and the period from the earliest records up to the search date (August 2016 for Q1, December 2016 for Q2). In July 2017, we conducted a supplementary bridge search to ensure that any newly published research was identified. The specific search strategies are available as Supporting Information (S2 Table).

We conducted “grey” literature searches of government agencies, professional organizations (e.g., medical societies and accrediting bodies), and other organizations that publish research (including the Cochrane Database of Systematic Reviews and the Joanna Briggs Institute) to identify other relevant studies, including conference proceedings and white papers. Using Web of Science, we reviewed references in and citations of our included articles to identify other potential relevant studies that were not identified in our electronic search.

### Study selection

Two investigators independently reviewed titles and abstracts, using a blinded process in the online program Covidence. We resolved discrepancies through consensus, erring on the side of inclusion for full-text review in cases of disagreement. We accepted all articles that did not include an abstract so that the full text of the article could be assessed for eligibility.

The same investigators independently reviewed the full text of articles for eligibility against pre-specified inclusion and exclusion criteria, using a blinded process in Covidence. We resolved discrepancies through consensus and consultation with a third investigator. The inclusion criteria for the full-text review was as follows: We included research studies that compared the impact of outpatient facility type (ASC vs. office/clinic) or specific facility characteristics on our designated outcomes (patient safety, patient experience and service availability) for procedures in non-hospital-affiliated outpatient settings. We excluded articles that summarized non-original research including commentaries and editorials, did not use a comparison group (e.g., studies of patient safety in a single setting), or measured only clinical outcomes (e.g., effectiveness of a procedure). We excluded studies conducted in hospital-affiliated outpatient settings, as these may be organized under the facility characteristics of the hospital.

### Quality assessment

Two investigators critically appraised the included studies using the ROBINS-I tool, which was developed by the Cochrane Collaboration to assess risk of bias in non-randomized studies [23]. The tool appraises the strengths and weaknesses of research across seven domains of bias—confounding, selection of participants into the study, classification of interventions, deviation from intended interventions, missing data, measurement of outcomes, selection of reported results—and offers signaling questions to guide the researcher in judging risk of bias within each domain. Risk of bias is categorized as low, moderate, serious or critical within each domain, and then assessed overall based on the most critical within-domain risk (e.g., a study is judged to be at serious risk of bias overall if it has been assessed at serious risk in at least one domain, but not at critical risk of bias in any domain).

### Data extraction and synthesis

We extracted data from the final sample of studies, including the data source, sample population, classification of exposure (i.e., outpatient facility type or specific facility factor), outcomes, analytic methods and relevant findings. One researcher extracted study-level data into evidence tables, and a second checked the data for accuracy. The ROBINS-I documentation notes that studies with critical risk of bias are “too problematic to provide any useful evidence and should not be included in any synthesis” [23] (p.4). Thus, we excluded studies judged to have critical risk of bias from our data extraction and synthesis. For studies that included multiple procedures in analyses, we extracted overall results rather than results by procedure. If overall results were not reported, we extracted results associated with the individual procedures. If multiple types of results were reported, we reported the most methodologically sound findings (e.g., results from regression models that controlled for confounding, rather than raw rates). We contacted authors for further information when statistical significance of key comparisons was not reported; however, authors often reported that information was unavailable years after publication.

Because of the great variation in study aims and outcomes, we did not quantitatively pool results across studies. Rather, we present results narratively by research question, noting study findings and highlighting any important limitations that might affect interpretation of results.

## Results

### Study selection process

**Fig 1:**
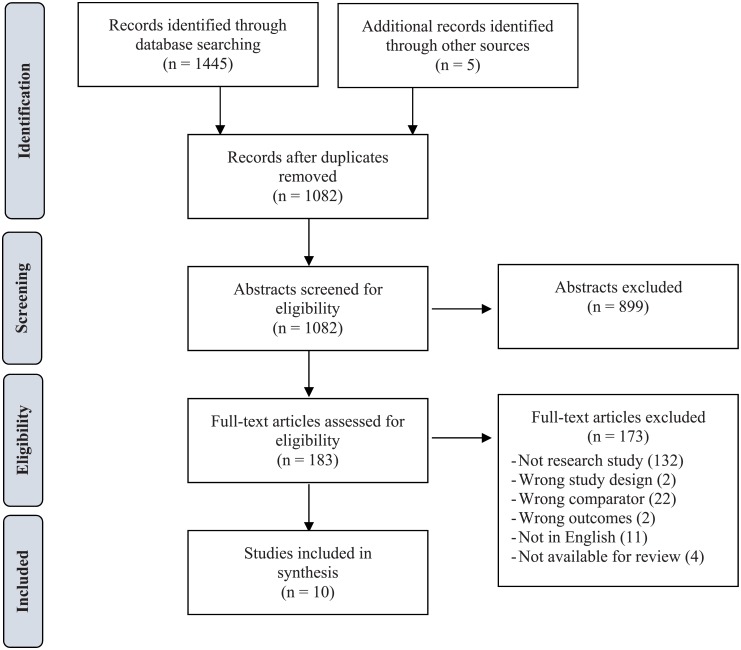
Study selection flow diagram, Q1 (effect of facility type).

**Fig 2:**
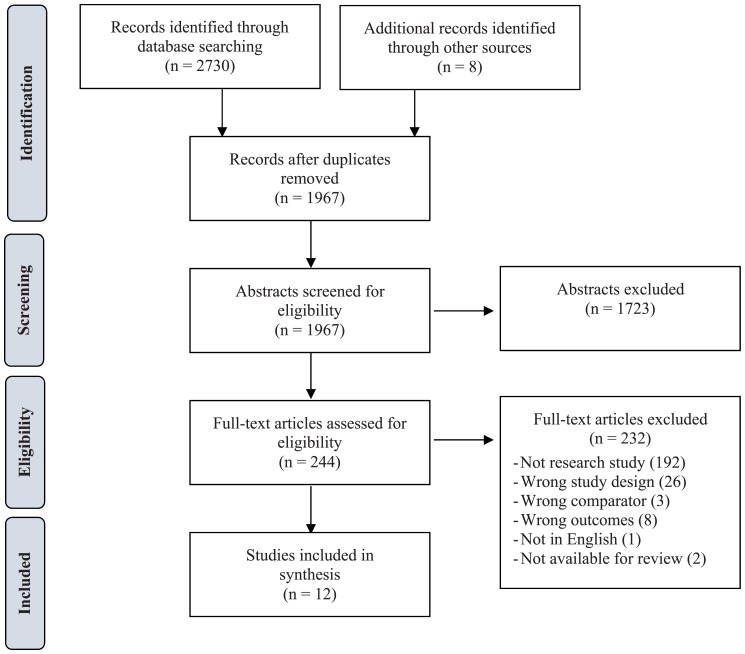
Study selection flow diagram, Q2 (effect of specific facility characteristics).

PRISMA flow diagrams, indicating the study selection process for each research question, are presented in Figs 1 and 2. For Q1 (Effect of Facility Type), the search strategy identified 1082 unduplicated articles for screening. We considered 183 eligible for full-text review and determined that 10 met criteria for inclusion in the review. For Q2 (Effect of Specific Facility Characteristics), the search strategy identified 1967 unduplicated articles for screening. We considered 244 eligible for full-text review and determined that 12 met criteria for inclusion in the review. In total, we identified 22 papers that met criteria for inclusion in the review.

### Study characteristics

The final sample of 22 studies are presented in Table 2. For Q1 (Effect of Facility Type), ten studies met inclusion criteria [11–15, 24–28]. The definitions of different facility types (“classification of exposure”) varied considerably across studies. Some studies compared accredited ASCs to accredited offices, whereas others compared accredited ASCs to non-accredited offices and ASCs. Other studies did not describe the criteria for classifying a facility as an ASC or office in detail. For Q2 (Effect of Specific Facility Characteristics), 12 studies met inclusion criteria [4–10, 20, 29–32]. Of these, eight studies examined the effect of facility accreditation, nine studies examined emergency response protocols, eight studies examined clinician qualifications, no studies examined physical plant characteristics, and one study examined other required facility policies.

**Table 2:** Studies of effect of facility type and specific facility characteristics on patient safety, patient experience and service availability for procedures in non-hospital-affiliated outpatient settings (N = 22).

	Author, Year	Research Question for Review	Data Source	Study Population	Medical Procedures	Classification of Exposure*	Outcome Type	Risk of Bias
**Q1. Effect of Facility Type**								
1	Colman & Joyce, 2011	Facility Type (ASC vs. Office)	State vital statistics	Texas residents having abortions at or after 16 weeks gestation in Texas and neighboring states, 2001–2006	Abortion	Before/after state ASC requirement law	Service Availability	Moderate
2	Fleisher et al., 2004	Facility Type (ASC vs. Office)	Medicare claims data	Nationally representative sample of Medicare beneficiaries undergoing surgical procedures, 1994–1999	Varied surgical	Accredited freestanding ASC vs. physician office/non-accredited ASC	Patient Safety	Moderate
3	Gupta et al., 2017	Facility Type (ASC vs. Office)	Voluntary private insurance claims data	Patients undergoing cosmetic surgery, prospectively enrolled in CosmetAssure insurance, 2008–2013	Cosmetic surgery	Accredited freestanding ASC vs. accredited office-based surgical suite	Patient Safety	Moderate
4	Hollingsworth et al., 2012	Facility Type (ASC vs. Office)	Medicare claims data	Nationally representative sample of Medicare beneficiaries undergoing outpatient procedures, 1998–2006	Urology	ASC vs. office	Patient Safety	Moderate
5	Housman et al., 2002	Facility Type (ASC vs. Office)	Provider survey	Members of American Society for Dermatologic Surgery who perform liposuction, reporting on patient cases, 1994–2000	Liposuction	Accredited ASC vs. non-accredited office	Patient Safety	Critical
6	Jani et al., 2016	Facility Type (ASC vs. Office)	Adverse event reporting	Patients undergoing outpatient surgical procedures with anesthesia, 2010–2014	Varied	Ambulatory facility (freestanding ASC or hospital-affiliated) vs. office practice	Patient Safety Patient Experience	Serious
7	Lee et al., 2013	Facility Type (ASC vs. Office)	Compiled media reports	Case reports of deaths from pediatric dental anesthesia, 1980–2011	Pediatric dentistry	ASC vs. office	Patient Safety	Critical
8	Rubino & Lukes, 2015	Facility Type (ASC vs. Office)	Patient survey	Randomized trial of women undergoing uterine polyp/ myoma removal	Uterine polyp/ myoma removal	Accredited ASC vs. accredited office	Patient Experience	Serious
9	Venkat et al., 2004	Facility Type (ASC vs. Office)	Adverse event reporting	Patients undergoing procedures in offices and ASCs in Florida, 2000–2003	Varied	ASC vs. office	Patient Safety	Serious
10	Vila et al., 2003	Facility Type (ASC vs. Office)	Adverse event reporting	Patients undergoing procedures in offices and ASCs in Florida, 2000–2002	Varied	ASC vs. office	Patient Safety	Critical
**Q2. Effect of Specific Facility Characteristics**								
11	Balkrishnan et al., 2003	Clinician Qualifications	Adverse event reporting	Adverse events following cosmetic surgery reported across state, 1999–2001	Cosmetic surgery	Board certification (Y/N)	Patient Safety	Critical
12	Boyle, 1996	Other Policies	Patient survey	Patients having surgery at single free-standing ASC, 1992 and 1994	Not reported	Before/after changes to facility procedures	Patient Experience	Critical
13	Clayman & Caffee, 2006	Facility Accreditation Emergency Response	Adverse event reporting	Patients having office-based surgery in Florida, 2000–2004	Varied	Facility accreditation (Y/N) Admitting privileges (Y/N) Board certification (Y/N)	Patient Safety	Critical
14	Clayman & Seagle, 2006	Facility Accreditation Emergency Response	Adverse event reporting	Patients having office-based surgery in Florida, 2000–2006	Varied	Facility accreditation (Y/N) Admitting privileges (Y/N) Board certification (Y/N)	Patient Safety	Critical
15	Coldiron, 2002	Facility Accreditation Clinician Qualifications	Adverse event reporting	Patients having office-based surgery in Florida, 2000–2002	Varied	Facility accreditation (Y/N) Admitting privileges (Y/N) Board certification (Y/N)	Patient Safety	Critical
16	Coldiron et al., 2004	Facility Accreditation Emergency Response Clinician Qualifications	Adverse event reporting	Patients having office-based surgery in Florida, 2000–2003	Varied	Facility accreditation (Y/N) Admitting privileges (Y/N) Board certification (Y/N)	Patient Safety	Critical
17	Coldiron et al., 2005	Facility Accreditation Emergency Response Clinician Qualifications	Adverse event reporting	Patients having office-based surgery in Florida, 2000–2004	Varied	Facility accreditation (Y/N) Admitting privileges (Y/N) Board certification (Y/N)	Patient Safety	Critical
18	Coldiron et al., 2008	Facility Accreditation Emergency Response Clinician Qualifications	Adverse event reporting	Patients having office-based surgery in Florida, 2000–2007	Varied	Facility accreditation (Y/N) Admitting privileges (Y/N) Board certification (Y/N)	Patient Safety	Critical
19	Gerdts et al., 2016	Emergency Response	Patient survey	Patients seeking abortion at clinics in 5 cities in Texas, 2014	Abortion	Nearest clinic closed or remained open after state admitting privileges law	Service Availability	Serious
20	Grossman et al., 2014	Emergency Response	Facility procedure data	Clinics providing abortion in Texas, 2012–2014	Abortion	Before/after state admitting privileges law	Service Availability	Serious
21	Menechemi et al., 2008	Facility Accreditation	Ambulatory surgery claims data	Ambulatory surgery and hospital discharge data on 5 procedures in Florida, 2004	Varied	Facility accreditation (Y/N)	Patient Safety	Moderate
22	Starling et al., 2012	Facility Accreditation Emergency Response Clinician Qualifications	Adverse event reporting	Patients having office-based surgery in Florida, 2000–2010, and Alabama, 2003–2009	Varied	Facility accreditation (Y/N) Admitting privileges (Y/N) Board certification (Y/N)	Patient Safety	Critical

* Classification of exposure, as defined by study authors

Most studies (19 of 22) involved retrospective analyses of existing data. Data sources varied across the 22 studies, including adverse event data collected through registries (11 studies), as well as administrative claims and discharge data (4 studies), prospective patient survey data (3 studies), and other sources. Nearly all articles (17 of 22) measured outcomes of patient safety (such as death, hospitalization, or emergency department visits). Few studies measured outcomes related to patient experience (3 studies) or service availability (3 studies).

### Study quality

For each study, risk of bias was assessed for each of the seven domains, and the overall risk of bias was based on the lowest domain assessment. Overall, zero studies had “low risk,” five had “moderate risk,” five had “serious risk,” and 12 had “critical risk” of bias. Overall results are presented in Table 2. Results by domain are included as Supporting Information (S3 Table).

Notable methodological challenges were found within the state of the literature. Eight of the 22 studies reported on the number and types of adverse events, often as a descriptive case series. These calculations lacked a denominator to estimate the proportion of procedures, patients or physicians experiencing adverse events in different facility settings or by specific facility requirement [4–9, 27, 29]. Other studies relied on combinations of datasets, where numerators and denominators were accessed from different sources, with conflicting results [11, 12]. Most studies did not control for potential confounders—such as patient demographic factors, patient health status, procedural invasiveness, or level of sedation—in statistical analyses [10–12, 24–26, 30, 31]. A few studies were hampered by poor response rates, unclear sampling strategies, the use of voluntary registries, which could have resulted in selection bias [25–27, 30]. A few studies, otherwise sound in design, included a large number of statistical tests without correcting for multiple comparisons, increasing the likelihood that statistically significant results are due to chance [26, 32].

Based on ROBINS-I guidelines, we excluded the 12 studies judged to have critical risk of bias from our data extraction. Among the remaining ten studies that met minimum quality criteria, seven examined effects of facility type (Q1) and three examined effects of specific facility characteristics (Q2).

### Effect of facility type

Seven studies met minimum quality criteria for Q1 (Table 3). Of these, five compared patient safety outcomes in the ASC and office setting. Across the five studies, one study reported mixed findings, three reported greater risk in the ASC, and one did not assess statistical significance. Across all 18 patient safety outcomes reported in the five studies, seven outcomes indicated greater risk in the ASC, one indicated lower risk in the ASC, six indicated no difference in risk by setting, and four did not assess the difference using statistical tests. Two of the seven studies reported on patient experience outcomes. One reported mixed findings, and the other found no statistical difference by ASC vs. office setting. One study examined the impact of a state-mandated ASC requirement, finding a decrease in service availability. Across all these studies, there is no consistent pattern to the results. The direction and statistical significance are typically consistent within studies, but are not consistent for outcomes across studies.

**Table 3:** Outcomes and results of research studies that met minimum quality criteria for Q1 (effect of facility type).

Author, Year	Outcomes	Procedures	Direction of Effect	Reported Results
Colman & Joyce, 2011	Number of in-state abortions at or after 16 weeks gestation among Texas residents	Abortion	Difference not assessed	Decrease in number of abortions one year after ASC law (3642 in 2003 vs. 446 in 2004). Not assessed for statistical significance.
	Number of out-of-state abortions at or after 16 weeks gestation among Texas residents	Abortion	Difference not assessed	Increase in number of abortions one year after ASC law (187 in 2003 vs. 736 in 2004). Not assessed for statistical significance.
	Abortion rate (abortions per 1000 women) at or after 16 weeks gestation	Abortion	Difference not assessed	Decrease in abortion rate three years after ASC law (0.78 in 2003 vs. 0.35 in 2006). Not assessed for statistical significance.
	Change in abortion rate (abortions per 1000 women) at or after 16 weeks gestation in Texas relative to Arkansas, Kansas, Oklahoma	Abortion	Greater decline in service availability in Texas compared to other states	Greater decrease in abortion rate in Texas relative to 3 comparator states among teens (β = -0.80, p < .05), adult women (β = -0.50, p < .01), and all women (β = -0.57, p < .01).
	Change in abortion rate (abortions per 1000 women) at or after 16 weeks gestation in Texas relative to 32 states	Abortion	Greater decline in service availability in Texas compared to other states	Greater decrease in abortion rate in Texas relative to 32 comparator states among all women (β = -0.55, p < .01).
Fleisher et al., 2004	Death	Varied	No difference in risk	Difference was not statistically significant. Numbers not reported.
	Emergency department visit within 7 days	Varied	Greater risk in ASC	Lower risk at office vs. ASC, controlling for other factors (OR = 0.71, CI: 0.61–0.84).
	Hospitalization within 7 days	Varied	Lowe risk in ASC	Greater risk at office vs. ASC, controlling for other factors (OR = 1.59, CI: 1.40–1.81).
Gupta et al., 2016	Major complication (defined as requiring hospital admission, emergency department visit, or reoperation within 30 days	Cosmetic surgery	Greater risk in ASC	Lower risk at office vs. ASC, controlling for other factors (OR = 0.67, CI: 0.59–0.77).
	Hematoma within 30 days	Cosmetic surgery	Greater risk in ASC	Lower risk at office vs. ASC, controlling for other factors (OR = 0.57, CI: 0.47–0.70).
	Infection within 30 days	Cosmetic surgery	Greater risk in ASC	Lower risk at office vs. ASC, controlling for other factors (OR = 0.71, CI: 0.55–0.92).
	Confirmed venous thromboembolism within 30 days	Cosmetic surgery	No difference in risk	Difference was not statistically significant. Numbers not reported.
	Suspected venous thromboembolism within 30 days	Cosmetic surgery	No difference in risk	Difference was not statistically significant. Numbers not reported.
	Pulmonary dysfunction within 30 days	Cosmetic surgery	No difference in risk	Difference was not statistically significant. Numbers not reported.
Hollingsworth et al., 2012	Death within 30 days	Urology	Difference in risk not assessed	No difference in risk at ASC or office, compared to hospital outpatient department. No statistical test comparing ASC to office.
	Same day hospitalization	Urology	Difference in risk not assessed	Greater risk at ASC vs. hospital outpatient department, controlling for other factors (OR = 6.96, CI: 4.44–10.90). Greater risk at office vs. hospital outpatient department, controlling for other factors (OR = 3.64, CI: 2.48–5.36). No statistical test comparing ASC to office.
	Hospitalization within 30 days	Urology	Difference in risk not assessed	No difference in risk at ASC or office, compared to hospital outpatient department. No statistical test comparing ASC to office.
	Postoperative complications within 30 days (identified using ICD-9 CM codes)	Urology	Difference in risk not assessed	Lower risk at ASC vs. hospital outpatient department, controlling for other factors (OR = 0.69, CI: 0.57–0.83). No significant difference in risk a t office vs. hospital outpatient department. No statistical test comparing ASC to office.
Jani et al., 2016	Inadequate postoperative pain control	Varied	Greater risk in ASC	Greater risk at ASC vs. office, not controlling for other factors (OR = 2.10, CI: 1.84–2.41).
	Postoperative nausea and vomiting (PONV)	Varied	Lower risk in ASC	Lower risk at ASC vs. office, not controlling for other factors (OR = 0.74, CI: 0.63–0.87).
	Eye injury	Varied	Greater risk in ASC	Greater risk at ASC vs. office, not controlling for other factors (OR = 9.05, CI: 1.27–64.42).
	Difficult airway	Varied	No difference in risk	No difference by facility type.
	Unexpected hospital admission (unspecified timeframe)	Varied	No difference in risk	No difference by facility type.
Rubino & Lukes, 2015	Patient “satisfied” or “very satisfied” at 12 months	Uterine polyp/myoma removal	No difference in patient experience	No difference by facility type.
	Patient would undergo treatment again if experienced similar symptoms	Uterine polyp/myoma removal	No difference in patient experience	No difference by facility type.
	Patient would recommend treatment to others with similar symptoms	Uterine polyp/myoma removal	No difference in patient experience	No difference by facility type.
Venkat et al., 2004	Mortality	Varied	Greater risk in ASC	Lower risk in office vs. ASC (RR: 0.45; CI: 0.24–0.85 or RR: 0.11; CI: 0.05–0.24, depending on data source for denominator).
	Adverse event	Varied	Greater risk in ASC	Lower risk in office vs. ASC (RR: 0.47; CI: 0.36–0.62 or RR: 0.05; CI: 0.03–0.09, depending on data source for denominator).

#### Summary of studies that met minimum quality criteria

Colman & Joyce (2011) used vital statistics data to assess the impact of a Texas state law requiring that abortions at or after 16 weeks gestation be performed in ASCs. Prior to the law, 95% of abortions at that phase of pregnancy were performed in physician offices or clinics; at the time, none met the requirements of ASCs. In the law’s first year, the number of abortions at or after 16 weeks gestation in Texas decreased by 88%, and the number in neighboring states among Texas residents increased fourfold. By three years later, the rate of abortions at or after 16 weeks gestation had decreased more than 50% (0.78 to 0.35 per 1000 women, in 2003 to 2006). In statistical models, the authors found greater declines in the rate of abortions at or after 16 weeks gestation in Texas than in comparable states (all p < .05). They conducted analyses to test alternative explanations, none of which conflicted with their conclusions. Minor methodological weaknesses of the study include not fully accounting for possible demographic changes over time and the selection of out-of-state data not including Georgia and Florida, which provide the bulk of later abortion procedures in the South.

Using a nationally representative sample of Medicare beneficiaries undergoing 16 varied outpatient surgical procedures, Fleisher et al. (2004) compared patient safety outcomes at accredited freestanding ASCs to physician offices and non-accredited ASCs. In regression models controlling for patient factors and type of surgical procedure, the authors found lower risk of emergency department visits (OR = 0.71) but higher risk of hospitalization (OR = 1.59) following surgery at offices compared to accredited ASCs. There was no statistically significant difference in risk of death. Separate analyses were reported for eight of 16 individual procedures, and risk of death or hospitalization was found to be greater at ASCs in seven of eight of these analyses. As noted by the authors, the interpretation of these results is confused by the combining of physician offices and non-accredited ASCs under the category “office” in Medicare claims data. The analysis was unable to control for type or duration of anesthesia use, and did not adjust statistical significance for the large number of statistical tests.

Gupta et al. (2016) relied on claims data from CosmetAssure, a voluntary private insurance for patients undergoing varied cosmetic surgery procedures at accredited ASCs and accredited office-based surgical suites (as well as hospital sites). CosmetAssure mandates that procedures be performed in accredited facilities, thus non-accredited offices or ASCs are not included. Risk of major complications (defined by the authors as those as requiring hospital admission, emergency department visit or reoperation) was significantly lower for patients in offices than in ASCs (RR = 0.67) after controlling for patient factors, procedure type and combined procedures. Similar results were found for some specific outcomes, including risk of hematoma or infection, but there was no difference in risk of VTE or pulmonary dysfunction by facility type. While analyses controlled for a number of potential confounders, the dataset did not include data on type or duration of anesthesia.

Hollingsworth et al. (2012) used a national sample of Medicare claims data to assess outcomes following 22 common urological procedures in freestanding ASCs, offices, and hospital outpatient departments (HOPD). The study found that the risk of same-day hospital admissions was significantly higher at ASCs and offices relative to HOPDs (OR = 6.96 and OR = 3.64, respectively), and that the risk of postoperative complications (as identified through ICD-9 CM diagnosis codes) was significantly lower at ASCs relative to HOPDs (OR = 0.69) but was not different at offices relative to HOPDs. However, the statistical models relied on the HOPD at the reference group and made no direct comparisons between the ASC and office. Thus, it is unclear if there were statistically significant differences in outcomes between the non-hospital-affiliated settings. Additionally, the analyses did not control for anesthesia use or specific procedure.

Using a voluntary quality improvement database of non-hospital-affiliated outpatient cases in which anesthesia was used, Jani et al. (2016) examined the impact of facility type on measures of patient safety and patient experience. Multiple procedure types were included, with outcomes reported overall and separately for each procedure. Overall, the study found no statistically significant differences in patients’ odds of difficult airway or hospital admission based on outpatient facility type. Rates of inadequate pain control was greater (OR = 2.10) and rates of post-operative nausea and vomiting were lower (OR = 0.74) for patients in the ASC relative to the office, which may reflect greater levels of sedation at the office. There were no statistically significant differences in difficult airway or hospitalization by facility type. These results are hampered by analyses that did not control for any potential confounders and the use of many statistical tests for each individual procedure and multiple outcomes for each procedure without correcting the statistical significance threshold to account for findings due to chance.

In a multi-center randomized trial of a hysteroscopic procedure for uterine polyps and myomas, Rubino & Lukes (2015), patients were randomized to treatment in an ASC or office setting. Among the 74 patients, one adverse event occurred at each facility setting, with neither case requiring hospitalization. In addition to treatment outcomes, the trial assessed patient satisfaction at 12 months. A greater proportion of patients at an ASC expressed satisfaction compared to those at an office (96.9% vs. 88.6%), which the authors attributed to greater levels of anesthesia used in the ASCs. However, this difference was not statistically significant (p = .07). There were no differences by facility type in the proportion of patient who would consider having the treatment again or would recommend the treatment to similar patients. Satisfaction scores were not controlled for other patient or procedural factors.

The study by Venkat et al. (2004) is presented as a direct response to Vila et al. (2013), which did not meet minimum quality criteria. Both rely on the mandatory reporting of adverse events in Florida and aim to determine the risk of mortality in physician offices compared with ASCs. The studies use different means to estimate the denominator—that is, the number of procedures in each setting in the state—to estimate risk. The findings of Vila et al., which indicated greater risk in offices, have been widely disputed for these calculations [8, 11]. In the updated analysis, Venkat et al. estimate higher adverse event rates and mortality rates in ASCs. The study estimates adverse event and mortality rates using two different data sources for the denominator, and the risk ratios vary considerably by data source. These calculations are also not adjusted for potential confounders, and therefore may still be at serious risk of bias.

### Effect of specific facility characteristics

Three studies met minimum quality criteria for Q2 (Table 4). One study addressed the effect of facility accreditation on patient safety outcomes, and two addressed the effect of emergency response protocols on service availability outcomes. No studies meeting minimum quality criteria addressed the impact of clinician qualifications, physical plant characteristics, or other facility policies. There is not enough research on each of the specific types of facility characteristics to draw conclusions across studies, although there is a suggestion that requiring abortion providers to have hospital admitting privileges may result in decreases in service availability for women seeking abortion.

**Table 4:** Outcomes and results of research studies that met minimum quality criteria for Q2 (effect of specific facility characteristics).

Data Source	Outcomes	Procedures	Direction of effect	Results
Menachemi et al., 2008	Hospitalization within 7 days	Arthroscopy	No difference in risk	No difference by for accredited vs. non-accredited ASCs.
	Hospitalization within 30 days	Arthroscopy	No difference in risk	No difference by for accredited vs. non-accredited ASCs.
	Hospitalization within 7 days	Cataract removal	No difference in risk	No difference by for accredited vs. non-accredited ASCs.
	Hospitalization within 30 days	Cataract removal	No difference in risk	No difference by for accredited vs. non-accredited ASCs.
	Hospitalization within 7 days	Colonoscopy	Lower risk for JC accredited vs. non-accredited. No difference in risk for AAAHC accredited vs. non-accredited.	Lower risk at JC accredited vs. non-accredited ASCs, controlling for other factors (OR = 0.891, CI: 0.799–0.993). No significant difference for AAAHC accredited vs. non-accredited ASCs.
	Hospitalization within 30 days	Colonoscopy	Lower risk for JC accredited vs. non-accredited. No difference in risk for AAAHC accredited vs. non-accredited.	Lower risk at JC accredited vs. non-accredited, controlling for other factors (OR = 0.906, CI: 0.850–0.966). No significant difference for AAAHC accredited vs. non-accredited ASCs.
	Hospitalization within 7 days	Upper Gastroendoscopy	No difference in risk	No difference by for accredited vs. non-accredited ASCs.
	Hospitalization within 30 days	Upper Gastroendoscopy	No difference in risk	No difference by for accredited vs. non-accredited ASCs.
	Hospitalization within 7 days	Prostate biopsy	No difference in risk	No difference by for accredited vs. non-accredited ASCs.
	Hospitalization within 30 days	Prostate biopsy	No difference in risk	No difference by for accredited vs. non-accredited ASCs.
Gerdts et al., 2016	Traveled more than 50 miles for care	Abortion	Decreased service availability if nearest clinic closed	Greater likelihood of traveling more than 50 miles if nearest clinic closed vs. remained open, controlling for other factors (43.8% vs. 9.6%, p < .001).
	Out-of-pocket expenses more than $100	Abortion	Decreased service availability if nearest clinic closed	Greater likelihood of out-of-pocket expenses more than $100 if nearest clinic closed vs. remained open, controlling for other factors (31.9% vs. 19.7%, p = .04).
	Overnight stay	Abortion	No difference in service availability	No difference in overnight stay if nearest clinic closed vs. remained open, controlling for other factors (16.0% vs. 5.1%, p = .07).
	Frustrated demand for medication abortion (preferred medication, but received aspiration)	Abortion	Decreased service availability if nearest clinic closed	Greater likelihood of frustrated demand for medication abortion if nearest clinic closed vs. remained open, controlling for other factors (36.8% vs. 21.8%, p = .003).
	Scheduled appointment later than preferred	Abortion	No difference in service availability	No difference in appointment delay if nearest clinic closed vs. remained open, controlling for other factors (45.7% vs. 45.4%, p = .94).
	Mean number of hardships experienced seeking care (scale 0–5)	Abortion	Decreased service availability if nearest clinic closed	Greater mean number of hardships if nearest clinic closed vs. remained open, controlling for other factors (1.67 vs. 0.90, p < .001).
	Patient reported “somewhat hard” or “very hard” to get to clinic	Abortion	Decreased service availability if nearest clinic closed	Greater likelihood of reporting “somewhat hard” or “very hard” to get to clinic nearest clinic closed vs. remained open, controlling for other factors (35.9% vs. 18.0%, p < .001).
	Gestational age ≥10 weeks at time of clinic visit	Abortion	No difference in service availability	No difference in gestational age if nearest clinic closed vs. remained open, controlling for other factors (30.2% vs. 26.4%, p = .83).
Grossman et al., 2014	Number of facilities providing abortion	Abortion	Difference not assessed	Decrease in number of abortion facilities from before to after the law (41 vs. 22). Not assessed for statistical significance.
	Annualized abortion rate, per 1000 women age 15–44	Abortion	Difference not assessed	Decrease in abortion rate from before to after the law (12.9 vs. 11.2 abortions per 1000 women age 15–44).
	Percent of all abortions using early medication abortion	Abortion	Decreased service availability after law	Decrease in percent of abortions using medication from before to after the law (28.1% vs. 9.7%, p < .001).
	Percent of all abortions using 1^st^ trimester surgical abortions	Abortion	Difference not assessed	Increase in percent of abortions as 1^st^ trimester from before to after the law (58.4% vs. 76.4%). Not assessed for statistical significance.
	Percent of all abortions using 2^nd^ trimester surgical abortions	Abortion	Decreased service availability after law	Increase in percent of abortions done in the second trimester from before to after the law (13.5% vs. 13.9%, p < .001).

JC = Joint Commission, AAAHC = Accreditation Association for Ambulatory Health Care

#### Summary of studies meeting minimum quality criteria

Menachemi et al. (2008) merged ambulatory surgery and hospital discharge data to compare hospital admissions for patients having procedures in accredited vs. non-accredited ASCs. Separate analyses were conducted for five common ambulatory surgical procedures, and compared results for ASCs accredited by the Accreditation Association for Ambulatory Health Care (AAAHC) or the Joint Commission, to those not independently accredited but overseen by the state regulatory agency. The authors found statistically greater risk of hospital admission for patients undergoing colonoscopy at non-accredited facilities compared to facilities accredited by the Joint Commission, controlling for patient and facility factors. No statistically significant differences were found for the other procedures or for those accredited by AAAHC. Given the high number of statistical tests conducted and lack of pattern in the results, the significant colonoscopy findings may be due to chance.

Two studies—Gerdts et al. (2016) and Grossman et al. (2014)—aimed to assess the impact on service availability of a 2013 Texas law requiring that abortion providers have admitting privileges at a local hospital. Grossman et al. found that the number of abortion facilities (41 to 22) and the annual abortion rate (12.9 to 11.2 abortions per 1000 women age 15–44) decreased from before to after the law was enacted; these were not assessed for statistical significance. There was a significant decrease in the percent of early medication abortions (28.1% vs. 9.7%, p < .001) and increase in the percent of abortions done in the second trimester (13.5% vs. 13.9%, p < .001). Surveying women seeking abortions, Gerdts et al. compared outcomes for women whose nearest clinic had closed or remained open following the enactment of the state law. They found greater distance traveled, out-of-pocket expenses, frustrated demand for medication abortion, number of hardships experienced, and patient reports that it was “somewhat hard” or “very hard” to reach the clinic (all p < .05) for women whose nearest clinic closed. There were no statistically significant differences in women needing to stay overnight prior to her abortion, scheduling an abortion later than her preference, or the gestational age of pregnancy. Both studies are methodologically sound policy evaluations, but challenged for the purposes of this review because the Texas law enacted other requirements (i.e., a requirement to follow an older medication abortion protocol) at the same time. It is therefore not possible to separate the specific effect of the admitting privileges requirement from other requirements.

## Discussion

In this systematic review, we examined the question of whether the type of outpatient facility or specific facility characteristics have an impact on patient safety, patient experience and availability of services. We found that the existing research literature is limited by methodological challenges, with many studies prone to biases that inhibit their utility in determining policy and practice. Across the studies of higher methodological quality, we found inconsistent results. Despite the methodological weaknesses and heterogeneity of study designs, it does appear that: 1) the existing evidence does not indicate a difference in patient safety for procedures performed in ASCs vs. physician offices; 2) requiring that abortions be performed in ASCs or that abortion providers have hospital admitting privileges appears to be associated with a decrease in service availability; and 3) there is insufficient research to draw conclusions from the existing body of research about the effect of specific facility characteristics on patient safety.

To some extent, these findings reflect an exploratory stage of research on this topic. The question of whether procedures should migrate out of the hospital has motivated research and practice considerations over the recent years [33, 34]. This focus is appropriate, as the potential harms of moving procedures that pose a risk of serious morbidity or adverse events such as hemorrhage, analgesic/anesthesia toxicity or over-sedation, or perforation from the inpatient to outpatient setting could be result in poor patient outcomes (e.g., hospitalization, additional surgical procedures, disability). In contrast, questions of which outpatient setting (i.e., ASC vs. office) is most appropriate for a given procedure already performed in outpatient settings or how those facility settings should be structured have been less pressing. As a result, it makes sense that most research has been exploratory, relying on case studies of adverse events from state registries [4–10, 29] or bringing together compilations of data sources [11, 12]. The limitations of these studies have been noted in more recent research (e.g., [14]. But such studies are important first steps in determining if there is a patient safety problem that may be due to facility type or facility characteristics and, if so, what intervention research might be needed to develop evidence-based solutions. We note that the research on patient safety in non-hospital-affiliated outpatient settings appears to be focused elsewhere, for example, on medication errors [35, 36], electronic health records [37–39] and office-based anesthesia [40, 41], rather than on questions of specific facility characteristics related to clinician qualifications, physical plant or other procedures. The notable exception is for facilities that provide abortion—a common outpatient procedure with a strong safety record in office/clinic settings [17–19]–which state legislatures have singled out, requiring them to comply with specific facility requirements [16, 22]. There is a body of research that has sought to predict or evaluate the impact of these requirements on abortion service availability. These studies indicate that the difficulty of compliance with Texas’ law resulted in the closure of about half of the state’s abortion facilities, increased burden on women seeking abortion, and delayed or prevented some women from having desired abortions [20, 24, 31, 42].

This systematic review makes clear that for procedures performed in non-hospital-affiliated outpatient settings, there is an absence of definitive research evidence about whether and what facility requirements may improve patient safety, as well as which, if any, of those requirements are able to improve patient safety without adversely affecting patient experience and service availability. Given the rarity of serious adverse events (e.g., death, hospitalization) following procedures in outpatient settings, insurance claims are likely the best source of data for future research, as they provide samples less affected by selection bias and include patient and procedure variables that can be controlled for in statistical analyses. In this review, the claims data analyses [13–15, 32] were least at risk of bias. However, there are other types of research evidence that did not meet the strict criteria of this systematic review that should be applied to questions of patient safety. This includes quality improvement databases developed by accreditation organizations [43–45] and professional associations (e.g., [46]), analyses of closed anesthesia malpractice claims analyses [47, 48], state-run registries [49], as well as best practices in office-based anesthesia [40, 41].

Research on procedures in outpatient settings needs to bring attention not just to concerns about safety, but also to outcomes of patient-centered care. This review makes clear that there is very little research on the impact of outpatient facility characteristics on patient experience and service availability. With the increasing recognition of the importance of care that is responsive to and respectful of patients’ preferences, needs and values [1], new studies would make strong contributions to the health care knowledge base by more thoroughly assessing patients’ experience with services. Validated measures of patient experience with health care provision, most notably the Consumer Assessment of Healthcare Providers and Systems (CAHPS) surveys [50, 51], are available for use in varied outpatient settings and encompass a broad view of patient experience across multiple domains. Qualitative methods have been used to understand patients’ perspective of health care services, including procedural care. For example, quantitative data has been combined with patient stories to create compelling evidence to evoke reflection and improvements within clinical teams [52]. Understanding the patient experience using qualitative methods has been shown to highlight potential solutions and opportunities to improve care [53].

In addition, new thinking is needed to study the impact of facility requirements on service availability, as facility requirements could limit access to care, as has been documented in relation to abortion [20, 24, 31, 42]. From a public health perspective, it is important to balance any possible improvements in patient safety with possible adverse health impacts of decreased service availability.

### Strengths and limitations

This study has important strengths, most notably its use of established systematic review methodology to identify relevant research, its formal risk of bias assessment to ensure that conclusions are drawn from the best available research, and its use of multidisciplinary experts to review the literature. Nonetheless, we may have missed relevant work in our search. Because the controlled vocabulary of our primary research databases do not include many facility-related terms, we relied on informal keywords that may have missed research that used other terminology. Other limitations result from variations in the identified studies. Because there is no standard definition of facility type that could be applied by authors, studies varied in their definitions and classifications of outpatient settings. Additionally, studies utilized datasets that varied in their populations, procedures and outcomes, which limited comparability across studies. As a result, we were not able to synthesize results or conduct meta-analyses across studies.

## Conclusions

In summary, we conclude that the existing research on the impact of facility type and facility-related characteristics on patient safety, patient experience and service availability for procedures in outpatient settings is limited. The existing evidence does not indicate a difference in patient safety for outpatient procedures performed in ASCs vs. physician offices. In addition, research on laws that have singled out abortion facilities with specific facility requirements appear to be associated with decreased availability of services. More and higher quality research is needed to determine if there is a public health problem to be addressed through facility regulation and, if so, which specific facility characteristics may result in consistent positive improvements to patient safety while not adversely affecting patient experience or service availability.

## Supporting information

S1 Table PRISMA checklist. (DOCX)

S2 Table Search strategy for systematic review. (DOCX)

S3 Table Risk of bias assessment for identified studies using ROBINS-I tool, by domain (N = 22). (DOCX)
