# Supplementary material for: The effect of facility characteristics on patient safety, patient experience, and service availability for procedures in non-hospital-affiliated outpatient settings: A systematic review
Source: PLoS One. 2018 Jan 5;13(1):e0190975. doi: 10.1371/journal.pone.0190975 (PMC5755935; doi:10.1371/journal.pone.0190975)
Supplement: S2 Table — (DOCX) [file pone.0190975.s002.docx]

**S2. Search strategy for systematic review.**

*Note: Strategy here is for EMBASE. Similar strategies used for PubMed and Web of Science*

**Q1. Effect of facility type**

| #1 | ‘ambulatory care’ OR 'outpatient care'/exp OR 'outpatient care' OR 'outpatient department'/exp OR 'outpatient department' OR 'ambulatory care facility' OR 'ambulatory care facilities' OR 'outpatient clinics' OR 'outpatient clinic' OR 'urgent care centers' OR 'urgent care center' OR 'urgent care clinics' OR 'urgent care clinic' OR 'family planning centers' OR 'family planning center' OR 'ambulatory health center' OR 'ambulatory health centers' OR 'abortion centers' OR 'abortion center' OR 'abortion clinics' OR 'abortion clinic' OR ‘abortion facility’ OR ‘abortion facilities’ OR 'free-standing clinic' OR 'free-standing clinics' OR 'outpatient surgery facilities' OR 'ambulatory setting' OR 'physician office' OR 'physician offices' OR 'physicians office' OR 'office procedure' OR 'office procedures' OR 'office-based surgery' OR 'office-based surgeries' OR 'office-based anesthesia' OR 'office based anesthetic' OR 'office based anesthetics' OR 'ambulatory surgery center' OR 'ambulatory surgery centers' OR 'ambulatory surgery centre' OR 'ambulatory surgery centres' OR ‘ambulatory surgical center’ OR ‘ambulatory surgical centers’ OR ‘ambulatory surgical centre’ OR ‘ambulatory surgical centres’ |
| --- | --- |
| #2 | 'treatment outcome'/exp OR 'patient experience'/exp OR 'patient experience' OR 'patient-centered care' OR 'patient-centred care' OR 'patient satisfaction'/exp OR ‘patient satisfaction’ OR 'patient safety'/exp OR ‘patient safety’ OR 'patient outcomes' OR 'treatment outcome' OR 'clinical effectiveness/exp' OR ‘clinical effectiveness’ OR 'patient-relevant outcome' OR 'patient relevant outcome' OR 'patient-relevant outcomes' OR 'clinical efficacy' OR 'treatment effectiveness' OR 'treatment efficacy' OR 'outcome assessment' OR 'outcome assessments' OR 'hospital admission' OR 'hospital admissions' OR 'adverse event' OR 'adverse events' OR 'adverse incident' OR 'adverse incidents' OR ‘morbidity’ OR ‘access’ OR ‘medication discrepancies’ |
| #3 | 'ambulatory surgery'/exp OR 'ambulatory surgery' OR 'surgicenter' OR 'surgicenters' |
| #4 | #1 AND #2 AND #3 AND [english]/lim |

**Q2. Effect of specific facility characteristics**

| #1 | ‘ambulatory care’ OR 'outpatient care'/exp OR 'outpatient care' OR 'outpatient department'/exp OR 'outpatient department' OR 'ambulatory care facility' OR 'ambulatory care facilities' OR 'outpatient clinics' OR 'outpatient clinic' OR 'urgent care centers' OR 'urgent care center' OR 'urgent care clinics' OR 'urgent care clinic' OR 'family planning centers' OR 'family planning center' OR 'ambulatory health center' OR 'ambulatory health centers' OR 'abortion centers' OR 'abortion center' OR 'abortion clinics' OR 'abortion clinic' OR ‘abortion facility’ OR ‘abortion facilities’ OR 'free-standing clinic' OR 'free-standing clinics' OR 'outpatient surgery facilities' OR 'ambulatory setting' OR 'physician office' OR 'physician offices' OR 'physicians office' OR 'office procedure' OR 'office procedures' OR 'office-based surgery' OR 'office-based surgeries' OR 'office-based anesthesia' OR 'office based anesthetic' OR 'office based anesthetics' OR 'ambulatory surgery center' OR 'ambulatory surgery centers' OR 'ambulatory surgery centre' OR 'ambulatory surgery centres' OR ‘ambulatory surgical center’ OR ‘ambulatory surgical centers’ OR ‘ambulatory surgical centre’ OR ‘ambulatory surgical centres’ |
| --- | --- |
| #2 | 'treatment outcome'/exp OR 'patient satisfaction'/exp OR ‘patient satisfaction’ OR 'patient safety'/exp OR ‘patient safety’ OR 'patient outcomes' OR 'treatment outcome' OR 'clinical effectiveness/exp' OR ‘clinical effectiveness’ OR 'patient-relevant outcome' OR 'patient relevant outcome' OR 'patient-relevant outcomes' OR 'clinical efficacy' OR 'treatment effectiveness' OR 'treatment efficacy' OR 'outcome assessment' OR 'outcome assessments' OR 'hospital admission' OR 'hospital admissions' OR 'adverse event' OR 'adverse events' OR 'adverse incident' OR 'adverse incidents' OR ‘morbidity’ OR ‘access’ OR ‘medication discrepancies’ |
| #3 | accreditation/exp OR accreditation OR accreditations OR accredited OR JCAHO OR AAAASF OR AAAHC |
| #4 | ‘admitting privileges’ OR ‘hospital-admitting privileges’ OR ‘emergency transfer’ OR ‘emergency transfers’ OR ‘transfer plan’ OR ‘transfer plans’ OR ‘transfer protocol’ OR ‘transfer protocols’ OR ‘emergency plan’ OR ‘emergency plans’ OR ‘emergency protocol’ OR ‘emergency protocols’ OR ‘patient transfer’ OR ‘patient transfers’ OR ‘transfer agreement’ OR ‘transfer agreements’ OR 'inter-facility' OR 'interfacility' OR 'hospital transfer' OR 'hospital transfers' |
| #5 | 'board certification' OR 'residency education'/exp OR 'certification'/exp OR certification OR certifications OR 'medical staff privileges' OR 'staffing ratio' OR 'nursing ratio' OR 'nurse ratio' OR 'nurse patient ratio'/exp OR 'nurse staffing' OR 'healthcare personnel/exp' OR 'ambulatory care nursing'/exp OR 'health care personnel management'/exp |
| #6 | ‘Procedure room’ OR ‘Operating room’ OR ‘Operating room’/exp OR ‘Sterilization room’ OR ‘Soiled instruments’ OR ‘Recovery room ‘ OR 'recovery room'/exp OR ‘Hall widths’ OR ‘Door widths’ OR ‘Design requirements’ OR ‘Layout’ OR ‘Emergency power’ OR ‘Power supply’ OR ‘Temperature requirements’ OR ‘Temperature control’ OR ‘Ventilation requirements’ OR ‘Air flow’ OR ‘Air exchanges’ OR ‘Air filtration’ OR ‘Air filters’ OR ‘National Fire Protection Association’ OR ‘Business occupancy’ OR ‘Health care occupancy’ OR ‘Facility design’ OR ‘Facility designs’ OR ‘Health facility environment’ OR ‘Built environment’ OR ‘Physical environment’ OR ‘Physical design’ OR ‘Facility design’ |
| #7 | 'quality control'/exp/mj OR 'quality control':ab,ti OR 'total quality management'/exp/mj OR 'total quality management':ab,ti OR 'peer review'/exp/mj OR 'peer review':ab,ti OR 'risk management'/exp/mj OR 'risk management':ab,ti OR 'risk assessment'/exp/mj OR 'risk assessment':ab,ti OR 'infection control'/exp/mj OR 'infection control':ab,ti OR 'infection prevention'/exp/mj OR 'infection prevention':ab,ti AND procedure OR procedures OR surgery OR surgeries |
| #8 | #1 AND #2 AND #3 AND [english]/lim |
| #9 | #1 AND #2 AND #4 AND [english]/lim |
| #10 | #1 AND #2 AND #5 AND [english]/lim |
| #11 | #1 AND #2 AND #6 AND [english]/lim |
| #12 | #1 AND #2 AND #7 AND [english]/lim |
