# Supplementary material for: The effect of facility characteristics on patient safety, patient experience, and service availability for procedures in non-hospital-affiliated outpatient settings: A systematic review
Source: PLoS One. 2018 Jan 5;13(1):e0190975. doi: 10.1371/journal.pone.0190975 (PMC5755935; doi:10.1371/journal.pone.0190975)
Supplement: S3 Table — (DOCX) [file pone.0190975.s003.docx]

**S3. Risk of bias assessment for identified studies using ROBINS-I tool, by domain (N=22).**

|  | **Author, Year** | **Confounding** | **Selection** | **Classification of Intervention** | **Deviation of Intervention** | **Missing Data** | **Outcome Measurement** | **Reporting** | **Overall Risk of Bias** |
| --- | --- | --- | --- | --- | --- | --- | --- | --- | --- |
| **Q1. Effect of Facility Type** | | | | | | | | | |
| 1 | Colman & Joyce, 2011 | Moderate | Low | Low | Low | Moderate | Moderate | Low | Moderate |
| 2 | Fleisher et al., 2004 | Moderate | Moderate | Low | Low | No Information | Low | Moderate | Moderate |
| 3 | Gupta et al., 2017 | Moderate | Low | Low | Low | Low | Moderate | Moderate | Moderate |
| 4 | Hollingsworth et al., 2012 | Moderate | Low | Low | Low | Low | Low | Moderate | Moderate |
| 5 | Housman et al., 2002 | Critical | Serious | Serious | Low | Moderate | Serious | Serious | Critical |
| 6 | Jani et al., 2016 | Serious | Moderate | Moderate | Low | No Information | Serious | Moderate | Serious |
| 7 | Lee et al., 2013 | Critical | Critical | Serious | Low | Critical | Low | Low | Critical |
| 8 | Rubino & Lukes, 2015 | Serious | Low | Moderate | Low | No information | Moderate | Moderate | Serious |
| 9 | Venkat et al., 2004 | Serious | Serious | Moderate | Low | Moderate | Serious | Moderate | Serious |
| 10 | Vila et al., 2003 | Serious | Critical | Moderate | Low | Critical | Serious | Low | Critical |

|  | **Author, Year** | **Confounding** | **Selection** | **Classification of Intervention** | **Deviation of Intervention** | **Missing Data** | **Outcome Measurement** | **Reporting** | **Overall Risk of Bias** |
| --- | --- | --- | --- | --- | --- | --- | --- | --- | --- |
| **Q2. Effect of Specific Facility Requirements** | | | | | | | | | |
| 11 | Balkrishnan et al., 2003 | Critical | Critical | Serious | Low | Critical | Serious | Low | Critical |
| 12 | Boyle, 1996 | Serious | Critical | Serious | Low | No information | Serious | Low | Critical |
| 13 | Clayman & Caffee, 2006 | Critical | Critical | Serious | Low | Critical | Moderate | Moderate | Critical |
| 14 | Clayman & Seagle, 2006 | Critical | Critical | Serious | Low | Critical | Moderate | Moderate | Critical |
| 15 | Coldiron, 2002 | Critical | Critical | Moderate | Low | Critical | Moderate | Moderate | Critical |
| 16 | Coldiron et al., 2004 | Critical | Critical | Moderate | Low | Critical | Moderate | Low | Critical |
| 17 | Coldiron et al., 2005 | Critical | Critical | Moderate | Low | Critical | Moderate | Low | Critical |
| 18 | Coldiron et al., 2008 | Critical | Critical | Moderate | Low | Critical | Moderate | Low | Critical |
| 19 | Gerdts et al., 2016 | Serious | Moderate | Serious | Low | Moderate | Low | Low | Serious |
| 20 | Grossman et al., 2014 | Serious | Low | Serious | Low | Moderate | Moderate | Low | Serious |
| 21 | Menachemi et al., 2008 | Moderate | Low | Low | Low | No information | Low | Moderate | Moderate |
| 22 | Starling et al., 2012 | Critical | Moderate | Moderate | Low | Critical | Moderate | Low | Critical |
